# A biophysical model of supercoiling dependent transcription predicts a structural aspect to gene regulation

**DOI:** 10.1186/s13628-016-0027-0

**Published:** 2016-02-06

**Authors:** Christopher H. Bohrer, Elijah Roberts

**Affiliations:** Department of Biophysics, Johns Hopkins University, 3400 N Charles St, Baltimore, USA

**Keywords:** Gene expression, Gyrase, Transcription, Bursting, Supercoiling, RNA polymerase

## Abstract

**Background:**

Transcription in *Escherichia coli* generates positive supercoiling in the DNA, which is relieved by the enzymatic activity of gyrase. Recently published experimental evidence suggests that transcription initiation and elongation are inhibited by the buildup of positive supercoiling. It has therefore been proposed that intermittent binding of gyrase plays a role in transcriptional bursting. Considering that transcription is one of the most fundamental cellular processes, it is desirable to be able to account for the buildup and release of positive supercoiling in models of transcription.

**Results:**

Here we present a detailed biophysical model of gene expression that incorporates the effects of supercoiling due to transcription. By directly linking the amount of positive supercoiling to the rate of transcription, the model predicts that highly transcribed genes’ mRNA distributions should substantially deviate from Poisson distributions, with enhanced density at low mRNA copy numbers. Additionally, the model predicts a high degree of correlation between expression levels of genes inside the same supercoiling domain.

**Conclusions:**

Our model, incorporating the supercoiling state of the gene, makes specific predictions that differ from previous models of gene expression. Genes in the same supercoiling domain influence the expression level of neighboring genes. Such structurally dependent regulation predicts correlations between genes in the same supercoiling domain. The topology of the chromosome therefore creates a higher level of gene regulation, which has broad implications for understanding the evolution and organization of bacterial genomes.

**Electronic supplementary material:**

The online version of this article (doi:10.1186/s13628-016-0027-0) contains supplementary material, which is available to authorized users.

## Background

The dynamics of gene expression in single cells has been studied extensively in the last 15 years, yielding new insights into the processes of transcription and translation [1–6]. Populations of cells are now known to exhibit a large degree of heterogeneity in both mRNA and protein expression levels [7]. The probabilistic nature of molecular reactions gives rise to the intrinsic component of this variation, while the differences between cells, such as the levels of RNAP, ribosomes, etc, produce the extrinsic component [8, 9]. Both types of noise contribute to the wide distributions of mRNA and proteins in a population [10–14]. But only extrinsic fluctuations are typically considered capable of introducing correlations into the expression levels of different genes within a single cell. The fluctuations and correlations can in principle be used to study the details of the underlying molecular processes [15].

One repeated theme when studying single cell gene expression is the occurrence of bursts during the production of mRNA and/or proteins. In particular, the production of mRNA has been shown to deviate from a simple birth-death process, instead occurring in transcriptional bursts [16, 17]. Transcriptional bursting gives rise to distributions with a Fano factor greater than one [14, 16, 18, 19]. Furthermore, it has been shown through single-molecule mRNA studies that the transcriptional bursting behavior is dependent on the expression levels and promoter architectures of the genes [14, 18]. The origin of these transcription bursts is still a subject of active inquiry and debate [14].

**Fig. 1:**
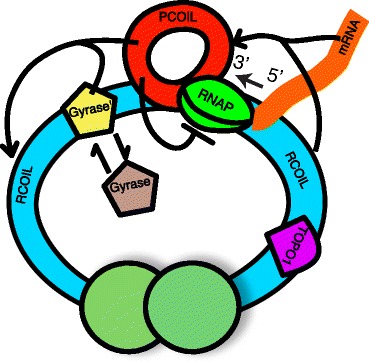
Positive Supercoiling (Pcoil) is produced when mRNA is transcribed. Pcoil inhibits the production of mRNA by reducing the initiation rate. In order to relieve Pcoil gyrase must bind (Gyrase’), which converts Pcoil into the “regular” state (Rcoil)

Recently, Chong et al. provided evidence supporting one contributing mechanism for transcriptional bursting in *E. coli* [20]. As RNA polymerase (RNAP) translocates along the DNA producing mRNA, positive supercoiling is generated downstream and negative supercoiling upstream of the enzyme complex [21]. In the absence of other factors, dissociation of RNAP would enable the positive and negative supercoils to resolve each other, leaving a zero net change in the supercoiling state. In *E. coli* there are two major factors when it comes to relieving supercoiling generated by transcription, topoisomerase I (Topo I) and gyrase. Topo I relieves negative supercoiling while gyrase relieves positive supercoiling. In *E. coli* Topo I has a higher activity than gyrase, as negative supercoiling can be very detrimental to the organism. This imbalance causes positive supercoiling to accumulate until gyrase binds and relieves the positive supercoiling [20]. The results of Chong et al. indicate that the build-up of positive supercoiling is one source of transcriptional bursting, where bursts occur when positive supercoiling inhibiting transcription of the gene is relieved, see Fig. 1.

Many models of gene expression have been proposed and studied, e.g. see [22–25]. However, none of these models account for the generation of positive supercoiling from transcription events. If this is part of the mechanism by which transcriptional bursting takes place, incorporating the accumulation of positive supercoiling in gene expression is vital in order to correctly describe the fluctuations and correlations of the system.

Here, we first develop a biophysical model to quantify the effect of supercoiling density on the transcription initiation rate. Then, using a simplified version of this model, we create a kinetic model of gene expression that directly accounts for the accumulation of supercoiling during transcription. When supercoiling is accounted for, our model predicts a change in the shape of mRNA distributions for genes with strong promoters, with an increase in low non-zero mRNA copy numbers. We then investigate the effect of having multiple genes in the same supercoiling domain and find a correlation in the expression of these genes. Having multiple genes in the same supercoiling domain also results in each gene’s expression influencing the expression of other genes in the same domain. These results not only provide insight as to how genes are expressed and regulated in bacteria, but also provides new directions for experimentally testing for the effects of domain coupled transcriptional bursting.

## Methods (model)

### Biophysical model for RNAP initiation with supercoiling

**Fig. 2:**
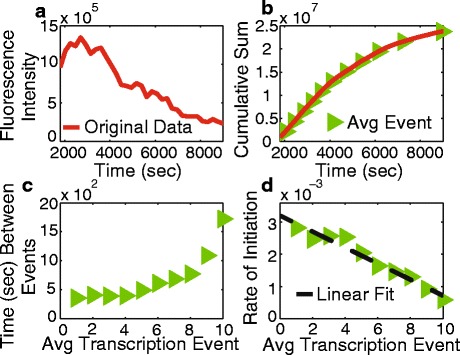
**a** Experimental data from [20] for T7 RNAP where the fluorescence intensity corresponds to the rate of transcript initiation in the absence of gyrase and the presence of Topo I. **b** The cumulative sum (*red line*) of the data in **a** corresponding to the total number of mRNA transcripts produced through time. Also shown are the times of average transcription events (*green triangles*) determined from the original data, see text. **c** The time between average transcription events. **d** The initiation rate by transcription event number (*green triangles*) and a linear fit (*dashed line*)

In order to produce mRNA, RNAP must bind and melt the DNA strands to allow an RNA-DNA hybrid to form before proceeding to elongation [18, 26]. This process necessitates maintaining the stability of the open complex long enough to form the DNA-RNA hybrid so RNAP can form an elongation complex [26, 27]. Recently, Chong et al. utilized an in vitro assay to demonstrate the effect of positive supercoiling on the rate of initiation for RNAP, both T7 RNAP and the *E. coli* RNAP. Their experiment monitored the production of mRNA from 160 individual molecules using an RNA-specific fluorescent dye. In the absence of gyrase, positive supercoiling accumulated, resulting in a decrease in the initiation rate of transcription. In their experiments, this manifested as a decrease in the fluorescence intensity with time as mRNA transcripts were being produced less frequently (Fig. 2 a, T7 RNAP). The cumulative sum of this data equals the total number of transcription events that had occurred by a given time (Fig. 2 b, red line). Using the reported intensity of a single mRNA transcript, 13.5×10^3^ [20], the average time it takes every template to produce an mRNA molecule can be calculated; this is termed an “average transcription event” (Fig. 2 b, green triangles). The time between successive transcription events shows the decline in initiation rate due to positive supercoil accumulation (Fig. 2 c). The decrease in the initiation rate with each average transcription event can be seen in Fig. 2 d and roughly decreases linearly.

In order to study why the initiation rate decreases in this manner we use the following kinetic model:

$$ \begin{aligned} \text{RNAP}+\text{DNA}& \underset{k_{\text{off}}}{\stackrel{k_{\text{on}}}{\rightleftharpoons}} \text{RNAP}:\text{DNA}_{\mathrm{c}}{\stackrel{k_{\text{cat}}}{\rightarrow}}\text{RNAP}: \text{DNA}_{\mathrm{o}}\\ &\quad\,\,{\stackrel{k_{\text{f}}}{\rightarrow}}\text{RNAP} + \text{mRNA}+\text{DNA,} \end{aligned} $$

where RNAP:DNA_c_ is the closed conformation and RNAP:DNA_o_ is the open conformations of RNAP. The rate *k*_cat_ is the rate at which the bound RNAP is able to transition to the open complex (melted DNA) and *k*_f_ is the rate at which the RNAP transitions to the stable elongation complex. These two rates are considered to be irreversible because of the small reverse rates [28, 29]. Using the steady state approximation and the assumption that the initial step in the initiation of transcription (*k*_cat_) is the rate limiting step [29], the kinetic model above results in the form of the classic Michaelis-Menten kinetic equation. The same Michaelis-Menten form has also been obtained for the steady-state approximation of more complicated kinetic models of transcription initiation [29–31]. In those studies, though, the definitions of the two constants *V*_max_ and *k*_*M*_ differ.

Considering the substrate copy number, specific promoter DNA, is much lower than the RNAP in a cell, the production rate of mRNA can then be approximated as:

(1) $$  V= k_{\text{cat}}/k_{M},\; \text{where} \; k_{M}=\frac{k_{\text{cat}}+k_{\text{off}}}{k_{\text{on}}}.  $$

The question of interest is how does supercoiling affect these different rates? It has been shown on linear pieces of DNA that the melting of the DNA in the promoter is a minor kinetic barrier [32]. However, positive supercoiling has been shown to increase the melting temperature of DNA and could cause the stability of the DNA to become influential [33]. Here we make the assumption that supercoiling only influences the *k*_off_ rate. This assumption is supported by experimental evidence that the binding affinity of T7 RNAP for the single stranded promoter sequence is greater and it dissociates slower with a single stranded promoter sequence [32, 34].

We do not rule out the possibility that the stability of the DNA could also influence the other kinetic rates in the model and the rates involved could differ depending on the particular RNAP. For example, considering that supercoiling of the DNA influences the amount of energy needed to melt the DNA, it also seems plausible that the supercoiling could affect the rate *k*_cat_. In the SI we repeat the below derivation for the *k*_cat_ rate and show that one obtains the same final result Additional file 1. Also, the existence of supercoiling sensitive promoters in *E. coli* for which positive supercoiling increases the transcription rate cast some doubt on such a simplifying assumption. However, additional experimental results would be needed to construct a more detailed model that differentiates how different RNAP states are influenced by supercoiling.

In the following, we make the assumption that the change in the free energy of the transition state for unbinding directly depends on the energy needed to melt the DNA of the promoter. In order to provide energetic insights as to how supercoiling would affect the DNA we utilized the statistical mechanical model of supercoiled DNA developed by Sen et al. [35, 36], which was built on the framework provided by Benham [37, 38]. This model showed close agreement with experimental results and was demonstrated over a wide range of supercoiling densities [33]. It should be noted that Benham has also utilized this model to develop a kinetic scheme for reactions with single stranded and double stranded DNA [39]. The free energy of having n melted base pairs, *n*_*j*_ junctions and a certain density of supercoiling, *σ* is:

$$ \begin{aligned} G(n, n_{j}, \sigma)&=n(\epsilon-T\Delta S)+\frac{n_{j}}{2}\times \epsilon_{o}+G_{s}(n,\sigma)+K_{b}T\\ &\quad\times ln [\!g(n, n_{j})],\\ G_{s}(n,\sigma)&=\frac{C \times N\left(\frac{n}{N}+\sigma\right)^{2}}{A^{2}[\!1+(\alpha-1)\frac{n}{N}]},\\ g(n, n_{j})&=\frac{N(N-n-1)!\!(n-1)!}{\left(N-n-\frac{n_{j}}{2}\right)!\left(n-\frac{n_{j}}{2}\right)!\left(\frac{n_{j}}{2}-1\right)!\left(\frac{n_{j}}{2}\right)!}. \end{aligned} $$

Here, *ΔS*=.024 kcal/(K mol) is the conformational entropy due to melting a base pair. *ε*=7.9 kcal/mol and *ε*_*o*_=2.5 kcal/mol are the base pairing and base stacking energies, respectively. The function *g*(*n*,*n*_*j*_) is the degeneracy factor with N total base pairs. $C=1638 \frac {\text {kcal}}{\text {mol} \times \text {rad}^{2}}$ and *α*=23.4 depend upon the bending and the torsional stiffness, respectively [36], and *A*=10.4 base pairs per rad^2^ for DNA in the relaxed state, see SI for derivation of supercoiling energy *G*_*s*_. We use the same parameters as obtained in [36].

Starting with n melted base pairs in a circular DNA loop of N base pairs the probability of having k melted base pairs in the promoter, consisting of Np base pairs, would follow a binomial distribution with a probability of successes equal to n/N. This is only valid when a lower fraction of the DNA is melted n/N <0.06, otherwise more than one melted base pair will be inside the same melted junction [35]. Here we assume that we are at room temperature and this assumption most likely holds. The probability of having a certain number of melted base pairs given the supercoiling density of the DNA would follow the Boltzmann distribution:

$$P(n | \sigma)=\frac{\sum_{n_{j}} e^{\frac{-G(n, n_{j}, \sigma)}{K_{b}T}}}{\sum_{n} \sum_{n_{j}} e^{\frac{-G(n, n_{j}, \sigma)}{K_{b}T}}}. $$

Then the change in the free energy barrier of the transition to a melted promoter at a certain *σ* is:

$$ \begin{aligned} \Delta G(\sigma)&=\sum_{n} \sum_{k}^{Np} P(n | \sigma) \times {Np \choose k} \left(\frac{n}{N}\right)^{k}\left(1-\frac{n} {N}\right)^{Np-k} \\ &\quad\times ((Np-k)(\epsilon)+\Delta G_{s}),\\ \Delta G_{s}&=G_{s}(n+Np-k,\sigma)-G_{s}(n,\sigma). \end{aligned} $$

The main effect the supercoiling density has is to alter the probabilities of having a certain amount of DNA melted, which influences the probability of having a melted base pair inside of the promoter region and the amount of energy needed to melt the DNA in the promoter region. Taking the difference of the transition state relative to the transition state at no supercoiling we obtain:

(2) $$  \Delta \Delta G(\sigma)=\Delta G(\sigma)-\Delta G(0).  $$

**Fig. 3:**
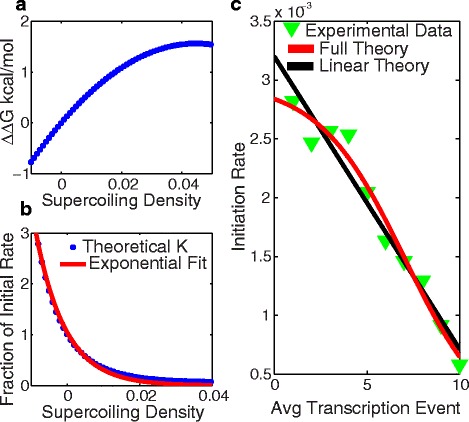
**a** The theoretical change in free energy needed to melt the base pairs of the promoter sequence by supercoiling density *σ*, from Eq. 2. **b** The change in the rate, K, by supercoiling density (*dots*) and a single exponential fit (*line*). **c** Transcription initiation rate vs the number of transcription events (*green triangles*) from experiment [20], the full theory Eq. 3 (*red line*) and the linear theory Eq. 4 (*black line*). The full theory had a fit R-square = 0.97 and the linear theory R-square = 0.96

Using the parameters obtained in [36], with N = 180 bp and Np = 8 bp, we numerically solved for the change in free energy; *ΔΔG*(*σ*), shown in Fig. 3 a. According to transition state theory the rate should depend upon *ΔΔG*(*σ*) according to: $k(\sigma)=k_{(0)} \times e^{-\frac {\Delta \Delta G(\sigma)}{K_{b}T}}$, where *k*_(0)_ is the initial rate with no supercoiling density. Numerical values for *k*(*σ*) are shown in Fig. 3 b.

In order to simplify further we approximate *k*(*σ*) as a simple exponential, demonstrated by the fit to the data generated by the model in Fig. 3 b, *k*(*σ*)=*e* ^−*wσ*^. This is only true if *ΔΔG*(*σ*) is linear with *σ*, which has been shown in numerous papers dating back to 1975 [40]. Plugging the exponential function into Eq. 1 and flipping the sign in the exponent for *k*_off_, where the negative in the exponent disappears because the open complex is trying to maintain the melted DNA, we obtain the following equation for the production rate of mRNA:

(3) $$  V(\sigma)=\frac{k_{\text{cat}} \times k_{\text{on}}}{k(o)_{\text{off}} \times e^{w \sigma}+k_{\text{cat}}} = \frac{k_{\text{on}}}{k' \times e^{w \sigma}+1},  $$

where *k*(*o*)_off_ is the initial off rate with no supercoiling, *w* is a constant and *k* ^′^=*k*(*o*)_off_/*k*_cat_. A fit to the experimental data from [20] with the 3 free parameters is shown in Fig. 3 c. The fit is merely illustrative here and the parameter values obtained (not shown) do not represent actual kinetic rates of T7 RNAP, because we are missing knowledge about the amount of supercoiling that results from one transcription event. The parameter values also have a large degree of uncertainly, due to the small number of data points.

By simplifying using an exponential we have combined the various energetic parameters that describe the biophysical characteristics of the system from the model into a single parameter *w*. Though we do not explicitly use the energetic parameters in the remainder of the paper, the full form of Eq. 2 could in principle be used to study supercoiling–transcription interactions in more biophysical detail, e.g., the sensitivity of different promoters to supercoiling based on base composition. We discuss such uses of Eq. 2 further in the SI.

Equation 3 can be further approximated using a Taylor series expansion, neglecting the higher terms of $\mathcal {O}(\sigma ^{2})$, which will be negligible, considering *σ*<<1 for *α*>>1 [36]. We then obtain a function for V linear in *σ*:

(4) $$  V(\sigma)=\frac{k_{\text{on}}}{k' \times e^{w \sigma}+1}\approx\frac{k_{\text{on}}}{k'+1}-\frac{k_{\text{on}} \times w \times k' \times \sigma}{(k'+1)^{2}}\,.  $$

This linear equation’s parameters have intuitive biological meaning: the y-intercept of the linear fit would then correspond to the production rate at zero supercoiling and the slope would determine how many times the loop of DNA could be transcribed before stalling.

Figure 3 c shows a comparison of the full and linear theories. There are many other factors that could be taken into consideration other than the melting of the DNA when it comes to the production rate of mRNA with supercoiling, but we consider this derivation a starting place to understand how the stability of supercoiled DNA can lead to the initiation rate decrease. We would like to particularly emphasize that the kinetic model of RNAP is a major simplification, considering that it does not include abortive initiation and the stability of the DNA could influence other kinetic rates. Also, the system may not necessarily start with a supercoiling density of zero, which would then lead to an even greater difference in the free energy to the transition state when supercoiling is accounted for. Due to the easy interpretation of the parameters extracted from the linear fit approximation, we use the linear model in the remainder of the study.

### Kinetic model for transcriptional bursting within a supercoiling domain

The accumulation of supercoiling due to transcription is primarily based on the “twin-supercoiled-domain model” of transcription [21]. Positive supercoiling in different domains builds up due to the absence of gyrase and the presence of Topo 1. Positive supercoiling from transcription has been shown to be a major factor in the build up of supercoiling and positive supercoiling has been shown to have a dramatic effect on the initiation rate of transcription in highly expressed genes [20, 41]. Therefore, it is critical that the buildup and release of positive supercoiling inside specific supercoiling domains be accounted for in modeling these processes.

To study the effect of supercoiling on mRNA and protein distributions, we combined our biophysical model, which describes the dependence of transcription initiation on the supercoiling state of the local DNA domain, with a kinetic model of gene expression. We based our kinetic model on a simple burst model of gene expression [24]. Burst models have been frequently used to model stochastic (probabilistic) gene expression [7, 24, 42, 43]. In the burst model, a gene is transcribed to produce mRNA, which is translated to produce protein. Both transcription and translation are first order processes without explicit RNAP or ribosome species. Both mRNA and protein decay also as first order processes. The burst model results in a Poisson distribution of mRNA molecules and a negative binomial distribution of proteins [10, 44]. Under some conditions, the negative binomial can be approximated by a gamma distribution [24], which is a two parameter distribution relating to the burst frequency and the burst size.

Here we present a modified gene expression model such that the transcription rate is linearly dependent on the amount of positive supercoiling that has accumulated in the local DNA domain, in accordance with our simplified biophysical model of transcription initiation. We first define two additional species *RCoil* and *PCoil* that track the amount of “regular”, the normal state, and positive supercoiling, respectively, inside the local domain. The sum of these two species is fixed and is denoted by *max*(*RCoil*). Production of a transcript converts one *RCoil* into a *PCoil*. Here, we assume an implicit fast relaxation of the corresponding negative supercoiling by Topo 1 [45]. Though, we do not rule out the possibility that other Topoisomerases may be contributing to the dynamics of these systems. The accumulation of *PCoil* linearly decreases the transcription rate of the DNA supercoiling domain according to reaction (1) in Table 1. The value of *max*(*RCoil*) is therefore equal to the number of times the DNA domain can be transcribed before transcription stalls.

**Table 1:** Kinetic model for gene expression with local supercoiling effects

Reaction		Propensity
(1)	*DNA*+*RCoil*→*DNA*+*PCoil*+*mRNA*	*a*_*o*_×*RCoil*
(2)	*mRNA*→0	*γ*×*mRNA*
(3)	*mRNA*→*Protein*	*b*_*o*_×*γ*×*mRNA*
(4)	*Protein*→0	*d*×*Protein*
(5)	*Gyrase*→*Gyrase* ^′^	*K*1
(6)	*Gyrase* ^′^→*Gyrase*	*K*2
(7)	*Gyrase* ^′^+*PCoil*→*Gyrase* ^′^+*RCoil*	*R*×*PCoil*

To model the relaxation of the positive supercoiling we introduce a gyrase binding site in the local DNA domain as an additional species that can either be empty *Gyrase* or bound with a gyrase molecule *Gyrase* ^′^. We assume a constant pool of free gyrase such that binding is pseudo first order. Gyrase unbinding follows first order kinetics. When gyrase is bound *PCoil* is converted to *Rcoil* with a rate constant *R* that is fast relative to the other rates in the system, such that when gyrase is bound the local domain is effectively always in the “regular” state. The complete model is shown in Table 1 and directly links the accumulation of supercoiling to the number of transcription events that have taken place since the last time that gyrase unbound.

**Fig. 4:**
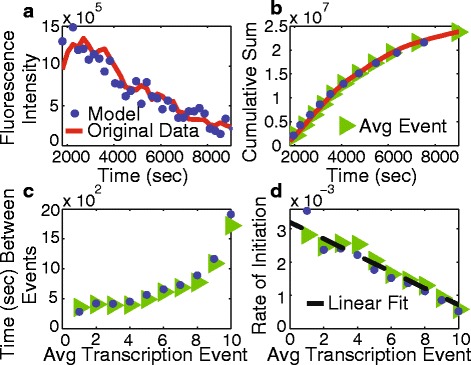
**a** Experimental data from [20] compared to the results from the model. Fluorescence intensity directly corresponds to transcription initiation rate. **b** The cumulative sum of the data in **a** compared to results from the model. **c** Comparison of the time between average transcription events between theory and experiment. **d** The initiation rate by transcription event number for the experimental data and for the model

To test our kinetic model, we simulated the in vitro experiment conducted by Chong et al. [20] by extracting two parameters from the linear fit in Fig. 2 d. The y-intercept of the linear fit in Fig. 2 d corresponds to the maximum initiation rate, *a*_*o*_×*max*(*RCoil*)=0.0032 sec^−1^. The x-intercept corresponds to the number of transcription events that can take place before stalling, *max*(*RCoil*)=13. All other rates and species were set to zero. We ran 160 simulations with a single gene in the supercoiling domain. The results of the simulations show good agreement with the original data despite the numerous approximations made in the derivation, shown in Fig. 4.

In order to take into consideration the stochastic nature of the reactions in Table 1 we used the Gillespie algorithm to simulate the model [46]. This was done assuming that the cell is a well stirred environment with no spatial constraints. These simulations where run using the program Lattice Microbes [47] with the rates normalized to the degradation rate of the protein, which is assumed to be on the order of cell division. In the remainder of this study, the rates for the equations in Table 1 were *γ*=50,*b*_*o*_=2,*d*=1,*K*1=10,*K*2=35,*R*=1000,*max*(*RCoil*)=4, except when specified in different specific situations.

The mRNA degradation rate, *γ*, was chosen so that the lifetime of the molecule would be short, ∼2 min. The value of max(RCoil) was set to 4, as opposed to using the fit value from the single-molecule in vitro experimental data shown earlier for the T7 RNAP. In *E. coli* it has been previously suggested that 4 rounds of complete transcription in a supercoiling domain could result in inhibiting transcription, due to the environment of the cell [20]. The rates for promoter strength, *a*_*o*_, and translation rate, *b*_*o*_, can take on a large range of values, we simply choose values that produced means that are physiologically relevant [7].

The range of rates for gyrase in the cell is a matter of debate and likely depends on many different factors. For instance, in vitro the dissociation constant has been measured to range from 0.2–0.5 nM for specific gyrase binding sites to 100 nM for weaker binding sites [20, 48, 49]. Though, what is taking place in vivo adds new factors to the system that need to be taken into consideration, e.g., there are endogenous inhibitors of gyrase [50, 51]. Using the Zero-spike model, discussed later, Chong et al. [20] estimated the ratio of the gyrase binding to unbinding rates for multiple genes in vivo using smFISH. They observed a range of values ∼0.1–4.5 demonstrating that the kinetic rates of gyrase vary greatly from gene to gene inside the cell. In our model, K1 was set to give an average rebinding time of $\frac {1}{10}$ of the cell doubling time (∼6 min for 1 h doubling) and then assuming a weak gyrase binding site with a ratio of K1/K2 = 0.28, K2 = 35.

## Results

### mRNA distribution

The probability distribution of the number of mRNA molecules per cell is an important quantity in gene regulation. Several previous studies have utilized smFISH to quantify the mRNA distributions of various genes [7, 14, 18, 20]. In order to study how the promoter strength of the gene influences the distribution of mRNA in our supercoiling model, we simulated one gene with a strong promoter and one gene with a weak promoter by setting *a*_*o*_= 100 and *a*_*o*_= 20, respectively. In the absence of supercoiling, these two promoter rates would result in Poisson distributions with a mean mRNA copy number of 8 for the strong and 0.4 for the weak promoter. To calculate the different distributions of mRNA generated by our supercoiling model, we simulated the equivalent of 4000 cells using the parameters specified above. Here, we ignore the protein part of the distribution, although we keep all rates relative to a protein degradation rate of 1.

**Fig. 5:**
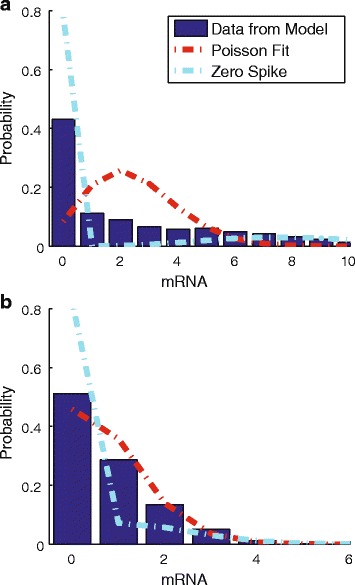
**a** The distribution of mRNA for a gene with a strong promoter (*blue bars*), a fit of the simulated data to a Poisson distribution (*red line*) and a fit to the zero-spike model (*cyan line*). **b** The same as in **a** for a gene with a weak promoter

Figure 5 a shows the mRNA distribution for the strong promoter with a weak gyrase binding site. Compared to a Poisson fit to the mRNA distribution, our supercoiling model spreads the density over a larger range of copy numbers. In particular, rather than being peaked at a non-zero copy number, our model predicts a large peak at zero copy number with a gradually decreasing density thereafter. Broadening of the mRNA distribution is also a characteristic of extrinsic noise and certain promoter architectures [14, 52], but here we focus only on the influence of supercoiling on the distributions.

Chong et al. [20] also developed a model to obtain a probability distribution of mRNA by incorporating gyrase binding and unbinding, the Poisson with Zero Spike (PZS) distribution. In their model the gene of interest switches on when gyrase is bound and immediately switches off when gyrase unbinds. However, the accumulation of supercoiling is not linked with the number of transcription events, thus the initiation rate does not decay as supercoiling accumulates. The distributions predicted by this model deviate substantially from the model proposed here. The PZS distribution obtained by using rates K1, K2, *a*_*o*_×*max*(*Rcoil*), and *γ* can also be seen in Fig. 5 a. The PZS distribution exhibits greatly enhanced density at zero copy number and has a distinct lack of density at low non-zero mRNA copy numbers. Our model and the PZS model converge when K1/K2 is extremely low.

When the promoter strength of the gene is low, the distribution from our model shows only minor deviations from a Poisson distribution, with a slight enhancement at zero copy number (Fig. 5 b). The corresponding PZS distribution again shows a greatly enhanced density at zero copy number.

**Fig. 6:**
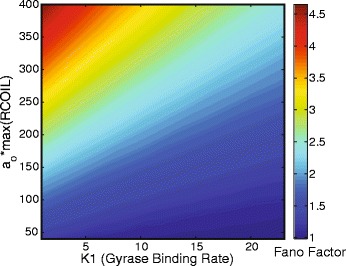
The Fano factor, variance/mean, of mRNA of a single gene inside a supercoiling domain with varying initiation rate *a*_*j*_ and gyrase binding affinity *K*1

The degree to which a distribution corresponds to a Poissonian can be quantified by the Fano factor, the variance divided by the mean, which is always one for a Poisson distribution. To study the influence of promoter strength and gyrase binding rate on the deviation of our mRNA distributions from Poissonian, we performed simulations across a wide range of these two parameters. Figure 6 shows how the Fano factor changes as these two parameters vary. As the binding affinity of gyrase decreases and the promoter strength increases, the Fano factor deviates further from that of a Poisson distribution. This overall trend is in agreement with experiments where genes with higher mean number of mRNA (due to stronger promoters) also have a higher Fano factor [14, 18].

The increase in Fano factor with mean mRNA copy number is typically ascribed to extrinsic noise. However, extrinsic noise shifts the Fano factor of highly expressed genes’ mRNA by broadening the distribution around the mean whereas in our model the Fano factor increases due to the expansion of the distribution toward higher mRNA copy numbers, while maintaining a significant density at zero and low copy numbers. Thus, the Fano factor by itself cannot be used to distinguish between genes with high variability from extrinsic noise or supercoiling, full distributions are necessary. Measured smFISH distributions likely contain noise contributions from both of these and other sources of noise and obtaining distributions from a large variety of native *E. coli* genes could help to disentangle the various contributions.

### Protein distributions compared to the burst model of gene expression

**Fig. 7:**
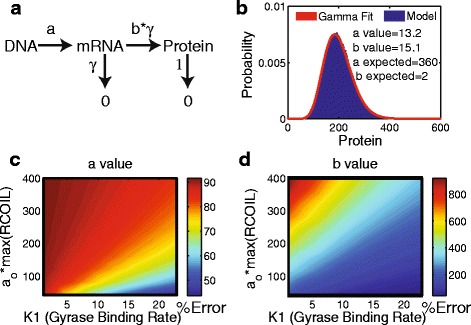
**a** The bursting model of gene expression. **b** The protein distribution generated from our model fit to a Gamma distribution. (Where the probability distribution above is for *K*1=10). **c**+**d** The percent error in the *a* and *b* values [ 100·(*fit*−*actual*)/*actual*] determined by fitting a gamma distribution to the data from the simulations

We next sought to compare the protein distributions produced by our supercoiling model against those produced by a typical model used for studying stochastic gene expression. The standard burst model of gene expression, shown schematically in Fig. 7 a, results in a negative binomial distribution of proteins [10]. Under conditions where the decay rate of mRNA is fast relative to that of proteins (*γ*>>1), as is typically the case in bacteria, the discrete negative binomial distribution can be approximated by the gamma distribution [10, 24]: $p(n)=\frac {n^{a-1}e^{-n/b}}{\Gamma (a)b^{a}}$ where *Γ* is the gamma function. The rates used in our model would correspond to the gamma distribution with *a*=*a*_*o*_·*max*(*RCoil*)/*d* and *b*=*b*_*o*_·*γ*/*γ*=*b*_*o*_.

Fitting protein abundance data to a gamma distribution gives an estimate for two key parameters from the model: *a*, the burst frequency, and *b*, the burst size. We wanted to study how well these two parameters could be estimated using a gamma distribution if the underlying data were generated by our kinetic model, which accounts for supercoiling induced transcriptional bursting.

We generated simulation data from our supercoiling model using the parameters *a*_*o*_=90, *max*(*RCoil*)=4, *b*=2, *γ*=50, *K*1=10, and *K*2=35. The stationary probability distribution for the protein is shown in Fig. 7 b. The distribution has the typical long tail seen in *E. coli* protein distribution data. We then fit our simulation data to a gamma distribution. Although the distribution appears to be well-described by a gamma distribution, the *a* and *b* parameters from the fit no longer correspond to the model parameters. The estimated *a* value was 13.2 and the estimated *b* value was 15.1, which are each approximately an order of magnitude from the correct values.

In a case where the gyrase binding site is predominantly occupied, i.e. when *K*1 is high and *K*2 is low, our model converges to the burst model. Likewise, when the initiation rate is comparable to the gyrase binding rate, the two models converge. To determine the parameter regions for which the two models give similar results, we performed a parameter scan of *K*1 and *a*_*o*_ and compared the model parameters versus the gamma fit parameters. Figure 7 c + d show the results of the comparison. We consistently find an underestimation of *a*, which is to be expected as *a* is an effective burst frequency while *a*_0_ is the basal transcription initiation rate. We also saw a consistent overestimation of *b*.

### Correlations between genes in supercoiling domains

The supercoiling domains in *E. coli* are thought to be loops roughly 10 kb in size [53]. The build-up of positive supercoiling in a local DNA domain affects not only the gene being transcribed, but all other genes in the domain. Here we assume that when a gene in a domain is transcribed the transcription of a neighboring gene does not cancel/enhance the supercoiling generated by its neighbor. However, if more than one gene in the supercoiling domain is being transcribed at the same time whether the genes are arranged in a codirectional or divergent pattern would be an important factor.

To study any correlations introduced into gene expression by this coupling, we modified our model to include multiple genes in a supercoiling domain. When a series of genes are present in same topological domain we refer to this as a linked domain. Promoters for all of the genes in a linked domain share the same supercoiling state, RCoil. To analyze expression correlations within a linked domain and to control for background correlations between independent genes, we constructed a system containing two independent supercoiling domains, each with five genes. We maintain the assumption that each DNA loop can be transcribed a total of four times before stalling, as discussed above. Since there are now five genes in each linked domain, we set *max*(*Rcoil*) to be 20 for each to allow them to be fully transcribed on average four times before stalling. Each of the two domains has a single and independent gyrase binding site, based on evidence that there is roughly one gyrase molecule for each loop in *E. coli* and gyrase is thought to have relatively specific binding sites [54].

**Fig. 8:**
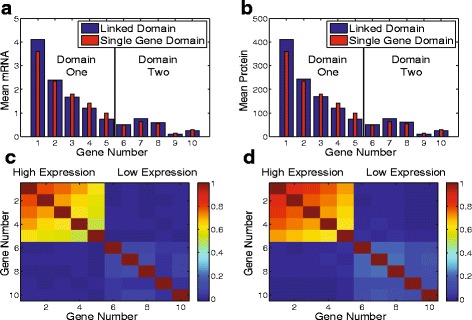
**a** The mean mRNA and **b** protein levels (*blue bars*) for genes from the simulations. Genes 1–5 share a supercoiling domain, while genes 6–10 share a supercoiling domain. A red bar shows the expression level of a gene if it was in its own supercoiling domain. **c** The correlation in the mRNA and **d** protein between the genes

We simulated a system in which the first linked domain contained genes with promoters ranging from strong to weak (genes 1−5) and the second linked domain contained genes with only weak promoters genes (6−10). The *a*_*o*_ values for the genes from 1 to 10 are 34, 20, 14, 10, 6, 2, 3, 2.4, 0.4, 1.0. We recorded the expression level of each gene over the course of the simulation. Figure 8 a + b show the mean expression levels of the mRNA and protein, respectively. Also shown are the expected values if each gene were in its own independent supercoiling domain. The mRNA and protein levels of relatively weak promoter genes 3–5, which reside in the supercoiling domain with the strong promoter genes, were significantly reduced. The weak promoter genes sharing supercoiling domain 2 were less perturbed.

Next, we calculated the pairwise correlations between mRNA and proteins for all of the genes, $p_{x,y}=\frac {cov(X,Y)}{\sigma _{X} \sigma _{Y}}$. The correlations are shown in Fig. 8 c + d. Both mRNA and protein of genes in the same linked domain showed correlated expression in individual cells and were only correlated with genes in the same linked domain. The correlation of the genes in individual cells was found to be dependent upon the promoter strength, where the presence of a gene with a strong promoter increases the correlation of all genes in the linked domain. The linked domain containing only genes with weak promoters exhibited significantly less correlation. In both cases the proteins showed slightly higher correlation than the mRNA. Weaker binding affinity for gyrase, K1/K2, for a linked domain also increased the correlation of the genes (data not shown).

### Negative regulation

**Fig. 9:**
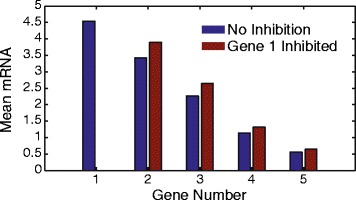
The mean mRNA level of the genes in a supercoiling domain with all genes expressed (*blue*) and when gene 1 is inhibited (*red*)

Finally, we investigated how regulating the promoter strength, *a*_*o*_, of a gene inside a linked domain would influence the expression level of other genes inside the domain. We performed simulations of a single linked domain containing five genes with decreasing promoter strength. The expression level of each gene in the linked domain was analyzed before and after the inhibition of gene 1, see Fig. 9. Inhibition of gene 1, which had the strongest promoter, led to an increase in the expression level of the other genes in the same domain.

## Conclusions

### Supercoiling build-up generates broad mRNA distributions

Our model linking supercoiling to transcription produces mRNA distributions that are noticeably different than those predicted by the burst and two-state models, as shown in Fig. 5. Of particular note is the enhanced probability at low (but greater than zero) copy numbers. Because transcription in our model gradually turns off, the distribution is spread over a wide range of low values, rather than being concentrated in a Poisson distribution for the high state and at zero for the low state. Anecdotally this agrees with some published mRNA distributions, e.g. [20], but a systematic survey must wait until genome-scale single-cell mRNA distribution data are available.

It has been shown that the Fano factor of the mRNA distributions is greater than one for highly transcribed genes [7, 14, 18]. This behavior is captured by our model, though we acknowledge that sources of extrinsic noise also contribute to this effect [14]. It is likely that the noisy distributions of mRNA observed in natural *E. coli* genes contain contributions from both supercoiling inhibited transcription and extrinsic sources of noise.

When analyzing the protein distributions produced by our model, we found that the *a* and *b* values deviated greatly from the predicted values of the burst model even though the distribution could be fit by a gamma distribution. If the fit to a gamma distribution is justified, the *a* value will correspond to the number of mRNA produced in a proteins lifetime and the *b* value will correspond to the number of proteins produced per mRNA transcript. Our model shows that when supercoiling is introduced the *a* and *b* values determined from the fit do not correctly represent the underlying processes of the system. Therefore, studies that rely on a physical interpretation of *a* and *b* may need to be adjusted.

Our results do not necessarily mean it is impossible to extract the underlying biological parameters from the distributions of mRNA and protein. Though we consider it to be beyond the scope of this paper, if the gyrase binding constants could be varied in a controlled manner and the distribution of mRNA could be obtained at different gyrase binding rates, then the parameters in our model could potentially be found through fitting. Even though there is no unique analytical formula for the distribution produced by our model, the parameters could be found by fitting to numerical solutions. Additionally, other known sources of noise, such as extrinsic noise, would also have to be incorporated into the model [14, 52]. This study provides a step toward a more detailed biophysical model of transcription during gene expression.

### Coordination of transcriptional bursts in neighboring genes

Correlation in the transcription of genes in bacteria has been previously reported. In [55], the expression levels of neighboring genes in *E. coli* were shown to be correlated and dependent on supercoiling. The authors concluded that expression levels were directly linked to the distribution of gyrase on the chromosome. Our model predicts this effect; gyrase has differing binding affinities for different supercoiling domains, which effectively controls the overall expression level of each gene in the same supercoiling domain. Other authors have reported that expression of an inducible reporter gene represses downstream neighboring genes [56]. Our model also exhibits this effect, as upregulation of one gene (or set of genes) in a supercoiling domain reduces expression of other genes in the domain.

Though correlation in the overall expression level of genes has been observed, our model predicts an additional degree of correlation, namely correlation in the transcriptional bursts of all genes in a supercoiling domain. Our model gives rise to correlation of not only protein abundance but also mRNA abundance of neighboring genes in a supercoiling domain, such as shown in Fig. 8. This correlation, coupled with the short lifetime of mRNA, means that bursts of mRNA molecules are also correlated in time between genes, i.e. neighboring genes are active and inactive in synchrony with each other. Such synchronization could be an important mechanism of transcriptional regulation in bacteria [57]. For instance, genes corresponding to specific functions are known to be located in similar areas on the genome. Synchronous expression of these genes would help to ensure all of the components are produced at the same time.

The correlation of clustered genes has been probed experimentally and clustering was not found to have a significant impact on the expression levels [58]. The authors proposed that any correlations due to gene clusters were washed out by the global extrinsic fluctuations. However, this was only done at a few specific locations in the chromosome and the effect of supercoiling may have been overlooked considering supercoiling not only affects the correlation in overall expression, but influences the correlation though time in the individual cells. In order to observe high correlation we found that the expression level of the genes inside the domain must be high enough to generate sufficient supercoiling to halt transcription before gyrase binds. We propose that mRNA smFISH of neighboring genes, carefully chosen for expression levels and to lie within a single supercoiling domain, would be an accurate assessment of whether correlation of transcription bursts occurs in *E. coli*.

### A structural level of gene regulation

Negative feedback has been proposed to be an important factor in controlling the stochastic nature of the biochemical reactions that take place in gene networks [59, 60]. Given that a transcription event of any gene inside the same supercoiling domain will increase the positive supercoiling felt by all genes inside the domain suggests, there is a higher level of negative regulation at the structural level for bacterial genomes. Every gene within the same domain would essentially negatively regulate the other genes inside that domain. This can be observed in Fig. 9, where the expression level of genes inside the same domain increase when a highly expressed gene inside the domain is inhibited. Such an effect could be essential for the proper stoichiometry of the gene products.

Having a built-in regulation network inside of the cell would help ensure the proper expression of certain genes without requiring extra energy for the production of transcription regulation factors specific for different genes. In this way, the expression level of genes in the same supercoiling domain would act as an intrinsic regulatory mechanism, providing yet another reason why the relative ordering of the chromosome is important. Such an effect would have implications in understanding the origin and widespread evolutionary conservation of operonal genome structure in Bacteria and Archaea.

## Availability of data and materials

The data supporting the conclusions of this article are publicly available with zenodo, which can be accessed through the following: https://zenodo.org/record/45167, https://zenodo.org/record/45168, https://zenodo.org/record/45178, https://zenodo.org/record/45196, https://zenodo.org/record/45198.

## Additional file

Additional file 1
**Supporting Information.** The SI of this paper is a brief derivations of the free energy of the DNA with supercoiling incorporated, an alternative derivation of the biophysical model with the supercoiling influencing the rate *k*_kat_ and an application of Eq. 2 from the biophysical model in future studies. (PDF 237 kb)
