# Supplementary material for: A biophysical model of supercoiling dependent transcription predicts a structural aspect to gene regulation
Source: BMC Biophys. 2016 Feb 6;9:2. doi: 10.1186/s13628-016-0027-0 (PMC4744432; doi:10.1186/s13628-016-0027-0)
Supplement: Additional file 1 — Supporting Information. The SI of this paper is a brief derivations of the free energy of the DNA with supercoiling incorporated, an alternative derivation of the biophysical model with the supercoiling influencing the rate k kat and an application of Eq. 2 from the biophysical model in future studies. (PDF 237 kb) [file 13628_2016_27_MOESM1_ESM.pdf]

Supporting Information for: A Biophysical Model of Transcription and Supercoiling Predicts a Structural Aspect to Gene Regulation

Chris Bohrer, Elijah Roberts

Derivation of Transition State Free Energy

This derivation is mostly taken from Sen et al. where an even more in depth explanation of the supercoiling free energy can be found. This derivation treats the DNA molecule as a "homopolynucleotide" in the shape of a circle where there is no base specificity. This DNA molecule has a total of N base pairs and a linking number equal to Lk, where a linking number is the number of base pairs per turn of the B form DNA. When a DNA molecule is supercoiled the linking number will change, ΔLk, which is just the difference between the relaxed linking number and the linking number with supercoiling. The number of base pairs per twist is equal to 10.4 and will be assigned to the letter "A". The twist rate is defined as $2\pi/A$ and the change in the twist rate in the helical regions will be assigned τ_h. Likewise, the twist rate in the melted regions will be τ_c. In order for the molecule to be stable the following equation must be obeyed:

$$c_h \cdot \tau_h = c_c \cdot \tau_c$$

Where c_h and c_c are defined as the "torsional stiffness constants." If we then examine the linking number of a partially melted DNA molecule, Lk', with n melted base pairs Lk' can be represented by the following equation:

$$Lk' = [(N-n)\tau_h + n \cdot \tau_c]/2\pi + (N-n)/A$$

Where the first term is the linking number due to supercoiling and the melted region and the second region is due to the regular melted regions. It is fundamental that the linking number has to remain constant throughout, Lk'=Lk, considering the molecule is a closed loop. Setting these two equations equal to each other and then solving the previous equations for τ_h and τ_c the following is obtained:

$$\tau_h = 2 \cdot \pi[\Delta Lk + n/A]/[N + (\alpha - 1)n]$$

and

$$\tau_c = 2\pi\alpha[\Delta Lk + n/A]/[N + (\alpha - 1)n] = \alpha\tau_h$$

Where $\alpha = c_h/c_c >> 1$ The supercoiling energy was derived in the studies by Benham and is beyond the scope of this SI for an in depth derivation. [2, 3, 4].

$$G_s = \frac{1}{2}(N - n)c_h \cdot \tau_h^2 + \frac{1}{2} n \cdot c_c \cdot \tau_c^2$$

Taking the previous equations for τ_h and τ_c and plugging them into the equation and using $\sigma = \Delta Lk \cdot A/N$, the supercoiling density, gives the free energy of supercoiling for the partially melted DNA molecule.

$$G_s(n, \sigma) = \frac{C \cdot N(\frac{n}{N} + \sigma)^2}{A^2[1 + (\alpha - 1)\frac{n}{N}]}$$

The total free energy of the DNA molecule can then be written as the following:

$$G(n, n_j, \sigma) = n(\epsilon - T\Delta S) + \frac{n_j}{2} \cdot \epsilon_o + \frac{C \cdot N(\frac{n}{N} + \sigma)^2}{A^2[1 + (\alpha - 1)\frac{n}{N}]} + K_b T \cdot lng(n, n_j)$$

$$g(n, n_j) = \frac{N(N - n - 1)!(n - 1)!}{(N - n - \frac{n_j}{2})!(n - \frac{n_j}{2})!(\frac{n_j}{2} - 1)!(\frac{n_j}{2})!}$$

This equation represents having all of the n melted base pairs being distributed into the n_j junctions. But here we are only interested in DNA molecules at room temperature, which corresponds to a small fraction of the molecule being melted. If we look at the average number of n_j for a certain amount of n melted base pairs we see that for small n the number of base pairs in each junction is very small [1]. This allows us to approximate the probability of having a certain number of melted base pairs inside of the promoter to be independent. This is where the binomial distribution results from in the text.

Derivation With Supercoiling Influencing the Rate k_{cat}

In the main text we have the change in free energy caused by supercoiling influencing the off rate in the kinetic model. Due to the fact that this assumption is a result from studies conducted with T7 RNAP, it is very possible that the free energy of the DNA could influence the transition to the open complex with the RNAP from *E. coli*. Here we show that we obtain the same results by modifying the rate k_{cat} .

Starting at Eq. 3 in the main text

$$V = \frac{k_{\text{cat}} \times k_{\text{on}}}{k_{\text{off}} + k_{\text{cat}}}.$$

If instead of associating the exponential with k_{off} we attached the exponential to the rate k_{cat}, this would result in the following equation:

$$V = \frac{k_{\text{cat}} \times e^{-w\sigma} \times k_{\text{on}}}{k_{\text{off}} + k_{\text{cat}} \times e^{-w\sigma}}.$$

Where there is a minus sign in the exponential because RNAP must melt the DNA to get over the free energy barrier. Interestingly, this is the exact same result as that found in the main text. If we simply simplify this equation it results in the following:

$$V = \frac{k_{\text{cat}} \times e^{-w\sigma} \times k_{\text{on}}}{k_{\text{off}} + k_{\text{cat}} \times e^{-w\sigma}} = \frac{k_{\text{on}}}{k' \times e^{w\sigma} + 1},$$

which is the exact same as Eq. 3 in the main text.

Sensitivity to Supercoiling is Dependent Upon the Biophysical Properties of the DNA

Here we discuss how Eq. 2 in the main text could be used with future research to predict which regions of the chromosome would have supercoiling sensitive gene expression. The main two parameters that could differ along the different regions of the chromosome are α and C. These parameters are related to the bending and torsional stiffness constants of the DNA [5]. C is only dependent upon the properties of the helical region, while α is dependent upon both the helical and coiled regions of the DNA. If future research investigating specific DNA has been done to determine the two parameters of various sections of the chromosome, Eq. 2 would be able to show how the free energy of the transition state varies with these different parameters. Hypothetically, if we assume we are looking at different DNA loops with similar C values and varying values of α we would obtain the data shown in Fig. S1. This shows the smaller α is the more sensitive to supercoiling the system will be. In the future one could potentially computationally determine these two parameters for different sections of DNA and predict which regions of the chromosome would be most affected by supercoiling.

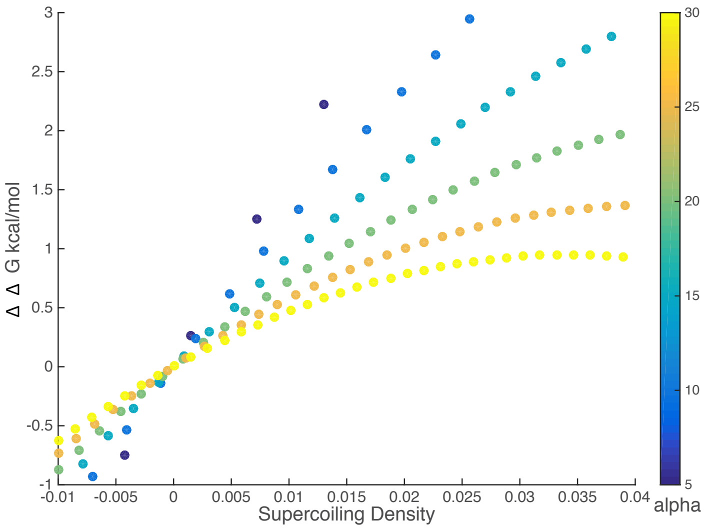

Figure S1: The change in free energy needed to melt the promoter of the DNA vs. the supercoiling density with varying α calculated from Eq. 2 in the main text with the same parameter values.

This also brings up the point that a transcription event may generate more positive supercoiling in different regions of the chromosome due to the biophysical properties of the DNA. If the specific biophysical parameters for different loops could be obtained experimentally and data describing how the initiation rate decreases with each transcription event could be obtained, one could use Eq. 2 to approximate the amount of supercoiling density generated with each transcription event. This could provide interesting information regarding the chromosome structure/dynamics.

References

[1] Sen, Srikanta, and Rabi Majumdar. "Statistical mechanical theory of melting transition in supercoiled DNA." Biopolymers 27.9 (1988): 1479-1489.

[2] Benham, Craig J. "Elastic model of supercoiling." Proceedings of the National Academy of Sciences 74.6 (1977): 2397-2401.

[3] Benham, Craig J. "Torsional stress and local denaturation in supercoiled DNA." Proceedings of the National Academy of Sciences 76.8 (1979): 3870-3874.

[4] Benham, Craig J. "Theoretical analysis of transitions between B-and Z-conformations in torsionally stressed DNA." Nature 286 (1980): 637-638.

[5] Sen, Srikanta, Ansuman Lahiri, and Rabi Majumdar. "Melting characteristics of highly supercoiled DNA." Biophysical Chemistry 42.3 (1992): 229-234.
